# PRAS: Predicting functional targets of RNA binding proteins based on CLIP-seq peaks

**DOI:** 10.1371/journal.pcbi.1007227

**Published:** 2019-08-19

**Authors:** Jianan Lin, Yuping Zhang, Wayne N. Frankel, Zhengqing Ouyang

**Affiliations:** 1 The Jackson Laboratory for Genomic Medicine, Farmington, Connecticut, United States of America; 2 Department of Biomedical Engineering, University of Connecticut, Storrs, Connecticut, United States of America; 3 Department of Statistics, University of Connecticut, Storrs, Connecticut, United States of America; 4 Institute for Systems Genomics, University of Connecticut, Storrs, Connecticut, United States of America; 5 Center for Quantitative Medicine, University of Connecticut, Farmington, Connecticut, United States of America; 6 Department of Genetics and Development and Institute for Genomic Medicine, Columbia University Medical Center, New York City, New York, United States of America; 7 Department of Genetics and Genome Sciences, University of Connecticut, Farmington, Connecticut, United States of America; Hebrew University of Jerusalem, ISRAEL

## Abstract

RNA-protein interaction plays important roles in post-transcriptional regulation. Recent advancements in cross-linking and immunoprecipitation followed by sequencing (CLIP-seq) technologies make it possible to detect the binding peaks of a given RNA binding protein (RBP) at transcriptome scale. However, it is still challenging to predict the functional consequences of RBP binding peaks. In this study, we propose the Protein-RNA Association Strength (PRAS), which integrates the intensities and positions of the binding peaks of RBPs for functional mRNA targets prediction. We illustrate the superiority of PRAS over existing approaches on predicting the functional targets of two related but divergent CELF (CUGBP, ELAV-like factor) RBPs in mouse brain and muscle. We also demonstrate the potential of PRAS for wide adoption by applying it to the enhanced CLIP-seq (eCLIP) datasets of 37 RNA decay related RBPs in two human cell lines. PRAS can be utilized to investigate any RBPs with available CLIP-seq peaks. PRAS is freely available at http://ouyanglab.jax.org/pras/.

## Author summary

It is important to identify the functional targets of RBPs, which are essential regulators in post-transcriptional processes. PRAS aims to predict RBP targets based on the intensities and positions of the binding peaks obtained from CLIP-seq studies. We demonstrate that PRAS score outperforms other existing methods not only in the prediction of the PCR-validated targets of the RBP CELF4, but also in the correlation with the global expression change induced by CELF proteins. The better performance of PRAS on a group of RBPs associated with RNA decay in comparison to the existing methods indicates its potential for large-scale applications in detecting functional targets. Leveraging the position information of the binding peaks, PRAS is a bridge linking peak-calling methods and the interpretation of RBPs’ biological functions, which strengthens the analysis of CLIP-seq data.

This is a *PLOS Computational Biology* Software paper.

## Introduction

RNA-binding proteins (RBPs) are essential in many post-transcriptional regulatory processes, such as alternative splicing, stability, localization and editing [1]. For example, RBP Quaking plays important roles in pre-mRNA splicing and mRNA export [2]; RBP HuR is an mRNA stability and splicing regulator [3]; RBP Ataxin-2 promotes mRNA stability and protein expression [4]. RBPs achieve their functions via binding to RNAs; therefore, it is of vital importance to study RNA-protein interaction. Cross-linking and immunoprecipitation followed by sequencing (CLIP-seq) approaches have been widely used to detect the binding peaks of RBPs at the transcriptome scale [5–9]. Thus, the examination of CLIP-seq peaks informs us of the functional targets of RBPs.

Existing computational approaches for analyzing CLIP-seq data focus on detecting RBP binding peaks [10–19] or differential RBP binding peaks between two different conditions [11, 14, 15, 20]. Computational methods for predicting the functional consequence of RBP binding peaks are less well-established [21–23]. Some studies suggest that the binding preferences of RBPs are associated with their specific functions. For example, HuR binding preferentially occurs close to the 3’ splicing site, which is consistent with its known function on alternative splicing [3]; Ataxin-2, an mRNA stability regulator, has a tendency to bind close to the polyadenylation site [4]. A recent study revealed that RBP TDP-43 regulates poly(A) site usage in a position-dependent way [22].

In this paper, we develop a new approach named Protein-RNA Association Strength (PRAS), which incorporates the intensity and positional information of CLIP-seq peaks to quantitate the association between an RBP and its targets. We apply PRAS to study two CUGBP ELAV-like family proteins, CELF4 and CELF1 with both CLIP and perturbation RNA-seq data available. CELF4 (also known as Brunol4) is expressed as an mRNA regulator in the central nervous system across species [24, 25]. The deficiency of CELF4 is associated with a complex neurobehavioral disorder including seizures and autism-like features in human [26, 27] and in mice [28]. iCLIP studies revealed that CELF4 preferentially binds, almost exclusively in 3’ untranslated regions (UTRs), to mRNAs encoding many important neurological functions, [29]. CELF1 is implicated in myotonic dystrophy [30]. CELF1 is highly expressed in early embryonic stages and are then down-regulated dramatically in skeletal muscle and the heart during development [31, 32]. CELF1 has been reported to promote transcript deadenylation and the abnormal up-regulation of its protein level could contribute to the myotonic dystrophy pathology [33, 34]. A more refined understanding of the functional targets of CELF RBPs is essential for understanding the impact of CELF in development and diseases, and may provide clues as to the mechanisms by which CELF impacts mRNA function. In addition, to demonstrate the robustness of PRAS, we examined its performance of detecting the functional targets in a large-scale collection of eCLIP data of RBPs in the integrated encyclopedia of DNA elements in the human genome (ENCODE). By applying PRAS to the eCLIP peaks of the RNA decay regulators, we demonstrate that PRAS outperforms other existing methods and also provide deeper understanding in the post-transcriptional regulation of these RBPs.

## Design and implementation

### The framework of PRAS

**Fig 1:**
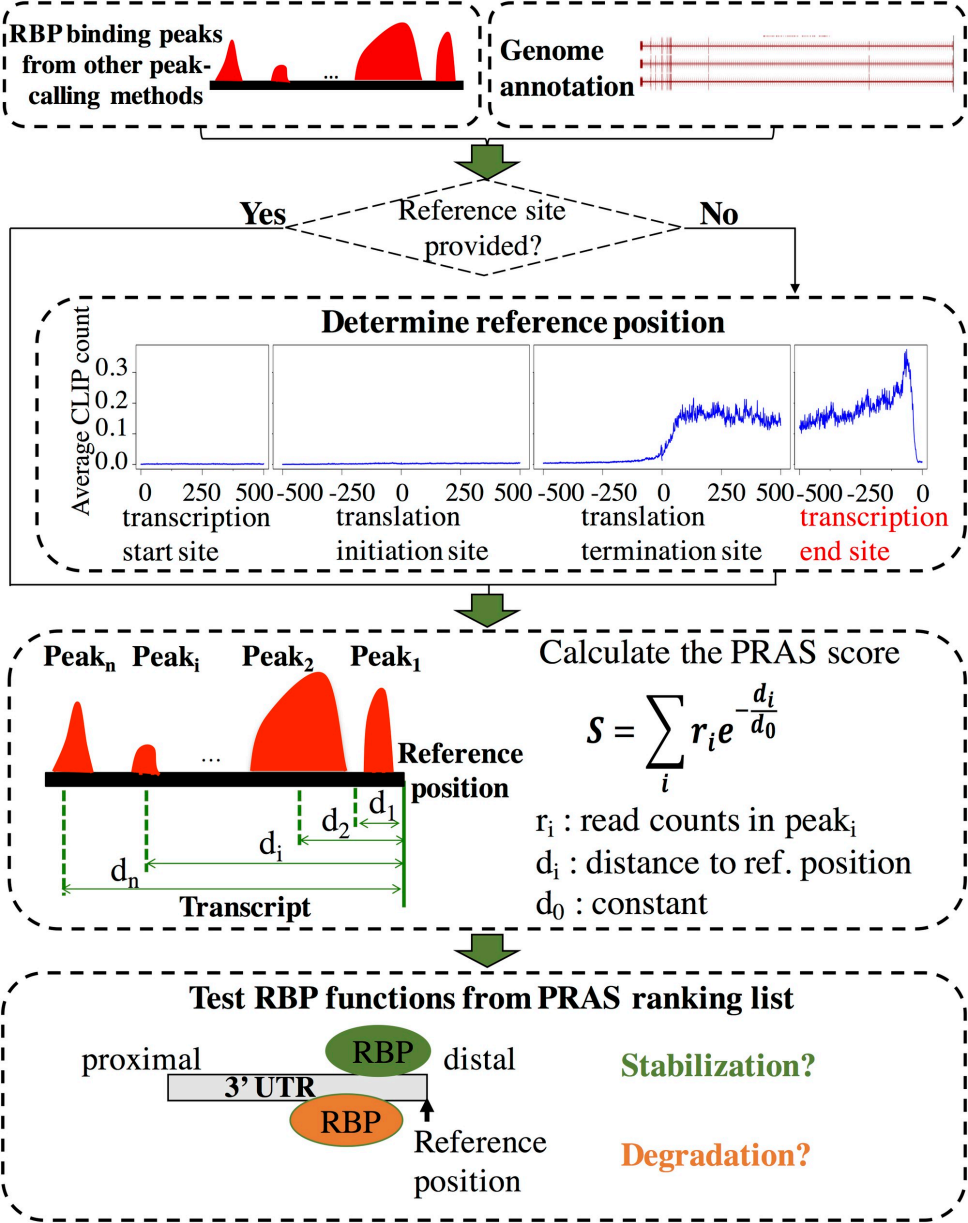
Flowchart of the PRAS pipeline. There are mainly three steps in calculating the PRAS scores. First, we merge the significant cross-linking sites as the binding peaks. Then, we use user-provided or automatically selected reference position and score each transcript based on both the intensities and the positions of the binding peaks. Finally, we rank the targets by PRAS and test RBP functions by independent datasets. The details of the PRAS calculation are described in the following section.

The basis of PRAS is to score a potential functional target of an RBP based on both the intensities and positions of its binding sites. Our pipeline of calculating PRAS is shown in Fig 1. First, given a CLIP-seq dataset, the significant cross-linking sites that are within a small interval of each other (default: 20 nt) are merged as RBP binding peaks. If the called binding peaks are provided, we will use them directly. Second, if a reference position is provided by the user based on known knowledge of the function of the RBP, PRAS will use it directly; if no reference position is given, PRAS will set it based on the RBP’s binding preference, e.g., the distal end of the 3’ UTR of the transcript (aka polyadenylation site). Finally, each transcript is scored as the sum of the intensities of the binding peaks weighted by the distances between the mid points of the binding peaks and the preselected reference position. All mRNAs are then ranked by the PRAS scores and can be tested for associations with functions.

### PRAS score calculation

As described in Fig 1, the PRAS score is based on the weighted sum of the intensities of the binding given detected CLIP-seq peaks. In the study that analyzed the interaction between DNA and proteins with ChIP-seq datasets, the exponential decay function was used to characterize the decreasing effects of a transcription factor binding peak on its targets with increasing distances [35]. Therefore, we here construct the score to describe the regulatory effect of an RBP on its targets in a similar way. Specifically, we define the PRAS score for an mRNA as:

$$S=\sum_{i}r_{i}e^{-d_{i}/d_{0}},$$ (1)

where *r*_*i*_ is the intensity (CLIP-seq read counts) of the *i*th peak cluster of the RBP, *d*_*i*_ is the distance (number of nucleotides) between the reference position and the *i*th peak cluster, and *d*_0_ is a constant. For both CELF4 and CELF1 in mouse, we set the reference position as the distal 3’ UTR and the constant *d*_0_ = 1000 nt. Note that *d*_0_ = 1000 nt is the default setting, but not a hard-set option in PRAS. For the RNA decay regulators in human, we set the constant *d*_0_ = 500 nt. The details of *d*_0_ estimation for RBPs in mouse and human are described in the Results and Discussions sections.

### PRAS implementation

PRAS is implemented in Python (version 2.7.14 or above) and R (version 3.3.2 or above) scripts and has minimum requirements for the inputs. To reformat the annotation file, PRAS takes use of gtfToGenePred, a toolkit from the UCSC Genome Browser [36]. PRAS also uses BEDTools [37] to efficiently obtain the overlapping between the binding sites and the annotation regions. The annotation file should be the Gene Transfer Format (GTF) format and the peak file (no special requirement for the peak caller) should be the Browser Extensible Data (BED) format as the required input files, which are both the standard file formats. Details of usage can be found on the instruction page of our website: http://ouyanglab.jax.org/pras/.

## Results and discussions

### PRAS score is a strong predictor of PCR-validated mRNA targets of CELF4

CELF4 is expressed in excitatory neurons of the adult mouse brain, from which iCLIP data are available [25, 28, 29]. We collected the significant cross-linking sites detected by iCount (http://icount.fri.uni-lj.si) with false discovery rate (FDR) less than or equal to 0.05. We conducted a metagene analysis involving all 9,193 mRNAs that are bound by CELF4 and noted an enrichment of iCLIP reads at the distal (3’ end) versus proximal (5’ end) 3’ UTR (S1 Fig). This preference suggests a potentially functional role of CELF4 binding close to the polyadenylation site.

**Fig 2:**
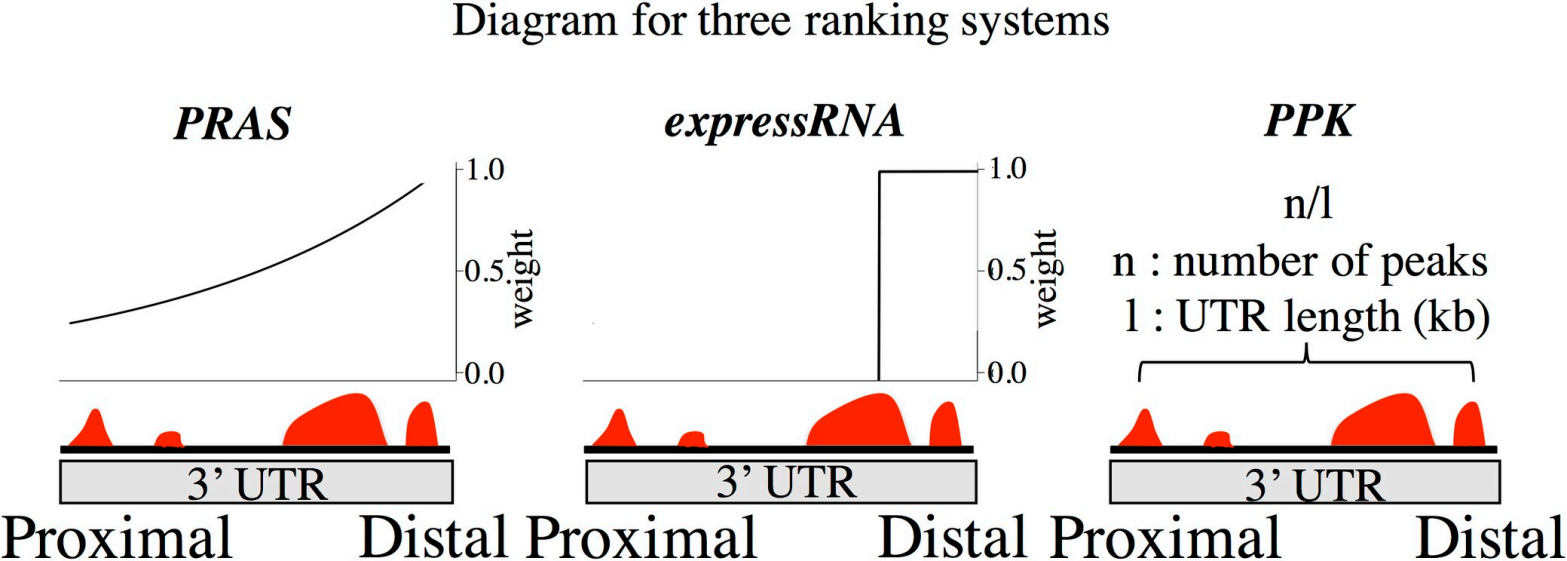
Diagram of three ranking methods. The three diagram shows how the score of genes are calculated for PRAS, expressRNA, and PPK, respectively. PRAS scores each transcript based on the sum of the intensities of the binding peaks weighted by an exponential decay function of the distances between the binding peaks and the reference position. expressRNA scores genes based on the total number of reads within 200 nt to the reference position. PPK calculates the number of peaks per kilobase.

We calculated the PRAS scores for CELF4 binding mRNAs with the polyadenylation site as the reference position, which gives the binding sites closer to the polyadenylation site higher weights. We estimated the decay parameter *d*_0_ in Eq (1) based on the strength of the peak intensity decay shown in S1 Fig. In detail, we defined the weighting formula as $w=e^{-d/d_{0}}$ according to Eq (1). The highest average peak density, 0.843, appears at 63 nt to the 3’ end of 3’ UTR and the average peak density at 1000 nt upstream to the 3’ end of the 3’ UTR is 0.285 (S1 Fig). We calculated *w* as the ratio between the average peak intensity at the 1000 nt upstream to the 3’ UTR and that of the 3' end of the 3’ UTR, which is 0.285/0.843 = 0.339. By plugging d = 937 nt (which is 1000 nt– 63 nt) and w = 0.339 into the weighting formula, we obtained the estimation of 866 nt for *d*_0_, which is approximately the default of 1000 nt. For comparison, we applied the expressRNA procedure of Rot et al. [22], which sums the number of reads in CLIP peaks within 200 nt upstream and downstream flanking the polyadenylation sites (Fig 2). We also applied the procedure in Wang et al. [34], which calculated the score as the number of significant CLIP peaks per kilobase (noted as PPK; Fig 2). Each of the three measurements ranks CELF4 binding mRNAs from high to low scores.

**Fig 3:**
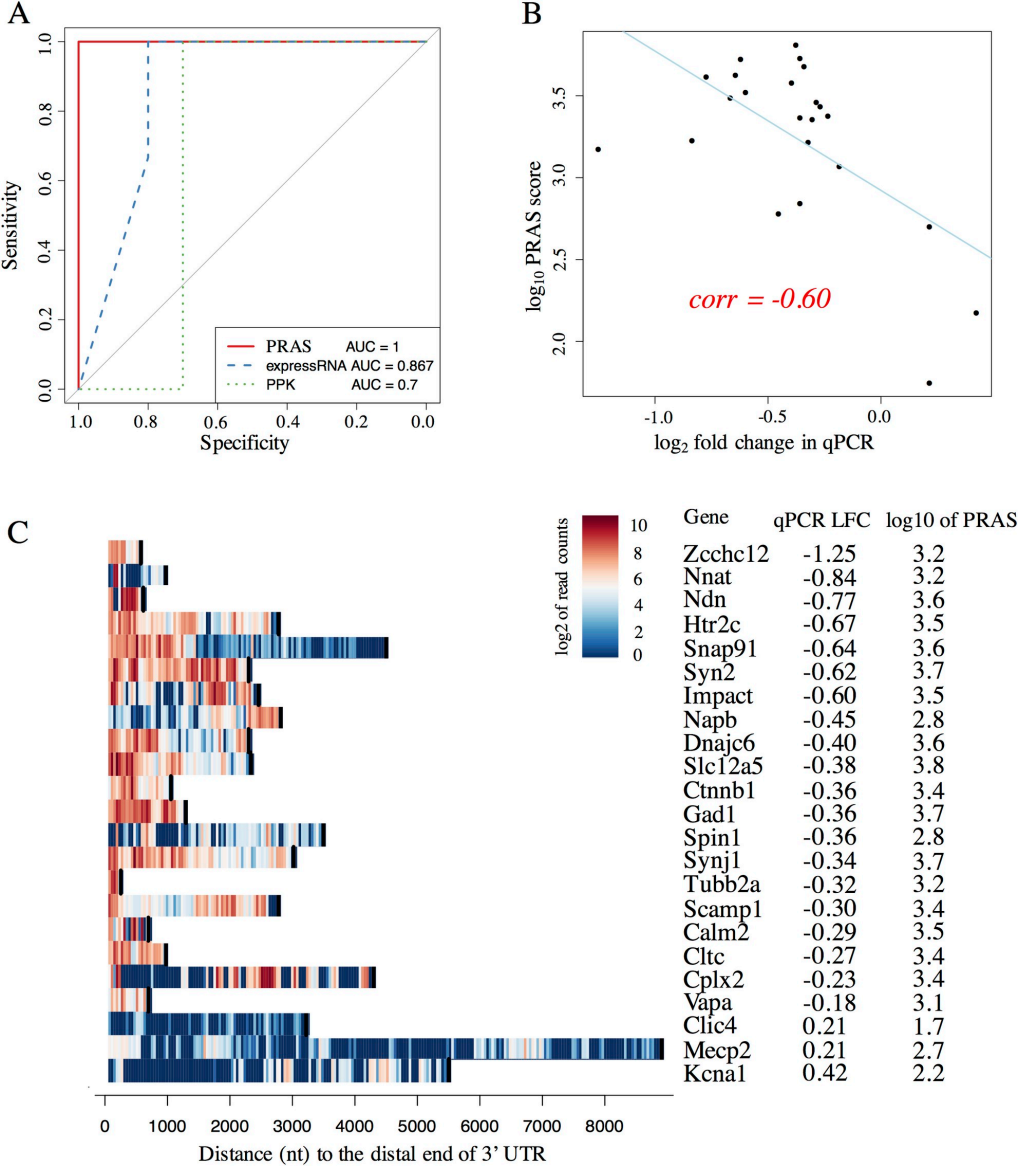
The qPCR-validated targets of CELF4. (A) The plot of ROC curves for PRAS, expressRNA, and PPK in the qPCR validated targets. The ROC analysis was done on the three methods’ scores and the expression change. The corresponding ROC curves for PRAS, expressRNA, and PPK are indicated by red solid, blue dashed, and green dotted lines, respectively. The AUC of the corresponding ROC curves are listed at the bottom of the plot. (B) The scatter plot of the PRAS score against the qPCR log fold change (LFC). The X-axis represents the log2 fold change in qPCR from the wild-type to Celf4 null mouse brain. The Y-axis shows the log10 of PRAS score. Each black dot represents a validated target by the qPCR. The regression line is highlighted in blue color. The Pearson’s correlation coefficient is indicated by the red text on the plot. (C) The heatmap of binding signals of the qPCR-validated targets. The X-axis represents the distance to the 3’ end of the 3’ UTR, and the Y-axis shows the genes in the validated list. The color shows the log2 of read counts of CELF4 iCLIP-seq within its significant peaks, where the warmer the color is the stronger the binding is. The black bars in each row shows the distance from the 5’ end of the 3’ UTR to the 3’ end of the 3’ UTR, which indicates the length of each 3’ UTR.

We then evaluated the performance of PRAS, expressRNA, and PPK on a list of known functional targets previously validated by qPCR in wild-type and *Celf4* null mouse brain, totaling 23 mRNAs [29] (see details in S1 Text). To investigate the ability of the three measurements to identify CELF4 functional targets, we performed receiver operating characteristic (ROC) analysis. We extracted the log fold change (LFC) of the qPCR values in *Celf4* null mouse brain over wild-type. The mRNAs with positive and negative LFCs were labelled as CELF4-degraded and CELF4-stabilized genes, respectively. The area under the curve (AUC) of the ROC curve was used to measure the prediction performance of the methods. We found that PRAS perfectly distinguished the PCR-validated CELF4-degraded and CELF4-stabilized genes (AUC = 1), outperforming expressRNA (AUC = 0.867) and PPK (AUC = 0.7) (Fig 3A). This result suggests that given CLIP peaks, PRAS has greater ability to capture the functional targets of CELF4 compared to expressRNA and PPK. In addition, we examined the quantitative relationship between the PRAS scores and the qPCR LFCs of these known targets. A negative Pearson’s correlation coefficient (-0.60) was obtained, suggesting that the more negative qPCR LFC a target has, the larger the PRAS score is (Fig 3B). The advantage of PRAS over expressRNA and PPK can be attributed to two factors. First, PRAS utilizes the binding bias of CELF4 towards the distal 3’ UTRs of its validated targets (Fig 3C). expressRNA partially utilizes this bias by considering the 200 nt flanking region around the polyadenylation site, whereas PPK does not consider the binding bias. Second, unlike expressRNA which only considers a fixed flanking region, PRAS considers all binding peaks, which decreases loss of important RBP binding sites. The analysis of the validated targets of CELF4 suggests the importance of binding near the polyadenylation sites as a potential factor on how it regulates gene expression. By applying different decay parameter *d*_0_ to PRAS, we found that PRAS obtained equally good performance over a reasonable range of *d*_0_s (S2A and S2B Fig). A *d*_0_ that falls out of certain range will decrease the performance of PRAS (S2A and S2B Fig), because a too small *d*_0_ can filter out the majority of iCLIP signals and a too large *d*_0_ approximates the uniform weighting. The stable performance of PRAS with *d*_0_ chosen around 1000nt shows the robustness of PRAS (S2 Fig).

### PRAS score correlates with global mRNA change induced by CELF RBPs

**Fig 4:**
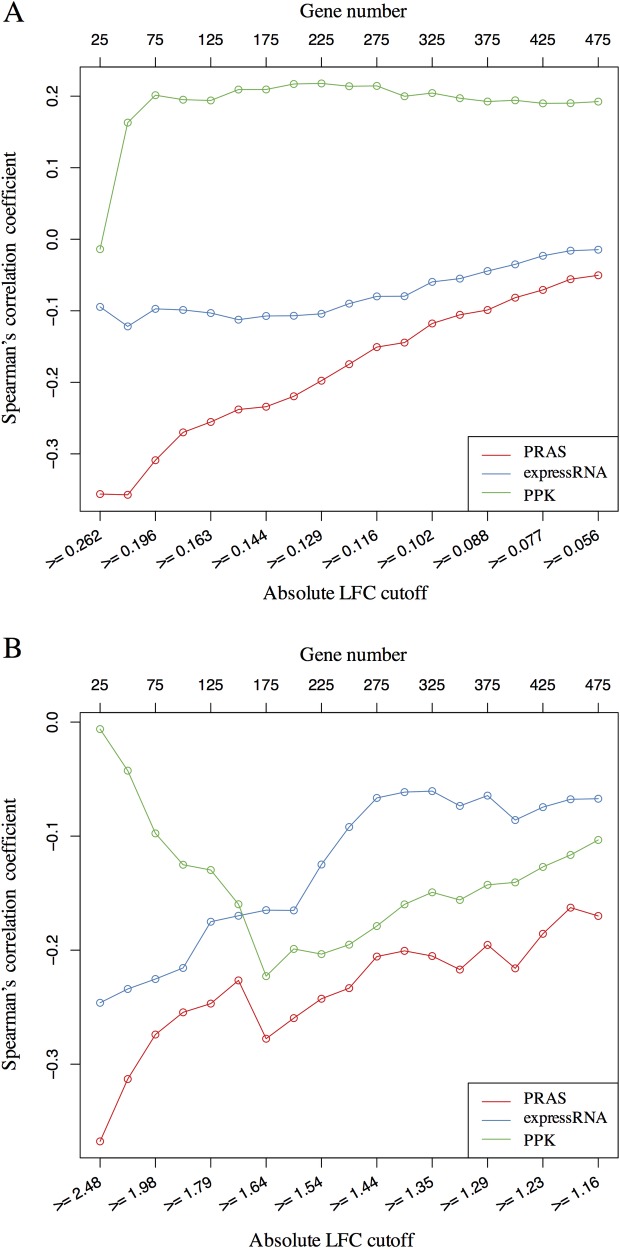
Correlation analysis between PRAS score and gene expression change. (A) The line-chart of Spearman’s correlation coefficient between the gene score and the gene expression LFC in the *Celf4*-regulated list. The lower X-axis represents the different cutoffs applied to extract the subset of genes, the upper X-axis represents the number of genes corresponding to the applied cutoffs, and the Y-axis shows the value of Spearman’s correlation coefficient. The corresponding curves for PRAS, expressRNA, and PPK are indicated by red, blue, and green lines, respectively. Each dot in the plot is for one subset of genes selected based on the absolute LFC cutoff. (B) Similar line-chart to A, but for the *Celf1*-regulated list. These two line-charts show that the higher ranked targets by PRAS have higher enrichment in the regulated lists comparing to the top ranked lists of expressRNA and PPK.

To assess the ability of PRAS to detect RBP functional targets in the entire transcriptome, we extracted the top 500 genes ranked by permutation test *p*-values in the differential expression test between the wild-type and *Celf4* null mouse brain based on existing microarray datasets [29]. We calculated the LFC for gene expression in *Celf4* null over wild-type mouse brain. The mRNAs have lower abundance (LFC < 0) in *Celf4* null genotype are more likely to be CELF4-stabilized targets, while the mRNAs with higher abundance (LFC > 0) in *Celf4* null brain were more likely to be CELF4-degraded targets. We sought to assess the ability of PRAS on capturing CELF4-stabilized vs. CELF4-degraded targets. Specifically, we first set a sequence of cutoffs as the quantiles (from 0.05 to 0.95 with step size as 0.05) of the distribution of the absolute value of the expression LFCs. Second, for each cutoff, we extracted a subset of genes whose absolute expression LFC is larger or equal to the cutoff. Finally, for each subset of potential CELF4 targets, we calculated the Spearman’s correlation coefficient between the expression LFCs and the PRAS scores, in which the magnitude and sign of the correlation reflect the association between the two. For comparison, we also applied the same correlation analysis to expressRNA and PPK ranking scores. Line-charts of the Spearman’s correlation coefficient of the three methods are shown in Fig 4A. We observed that the more stringent the expression LFC cutoff for the gene subset was set, the stronger the negative correlation between the PRAS score and the expression LFC was obtained, which suggests that PRAS is more powerful in capturing more reliable CELF4-stabilized targets. In addition, the expressRNA score is less correlated with the expression LFC, and the direction of the correlation between the PPK score and the expression LFC flips at different cutoffs. The results suggest that PRAS has greater ability to select the regulated mRNA targets compared to expressRNA and PPK.

We also extracted the top 500 genes ranked by their adjusted *p*-values in the differential expression test between the wild-type and *Celf1* over-expression in mouse muscle based on published RNA-seq datasets [34]. In this dataset, mRNAs that have higher abundance upon *Celf1* over-expression (LFC > 0) are more likely to be CELF1-stabilized targets while those that have lower abundance upon *Celf1* over-expression (LFC < 0) were more likely to be CELF1-degraded targets. We evaluated the performance of the three aforementioned methods using the same analysis as with CELF4. We used the 3’ end of the 3’ UTR as the reference site in PRAS to rank the mRNA targets based on the reported binding preference of CELF1 [34]. PRAS has a stronger negative correlation with the expression LFC compared to expressRNA and PPK for each subset of the potential CELF1 targets (Fig 4B). These results suggest that PRAS is more powerful in capturing the reliable CELF1-degraded targets, consistent with the main regulatory function of CELF1 [34].

Next, we used different reference sites in PRAS for scoring functional targets of CELF4 and CELF1 in order to examine the effect of the reference site selection. We scored the targets of CELF4 using the 5’ end of the 3’UTR as the reference site in PRAS (PRAS 5’) and did a similar correlation analysis as above. We observed that the PRAS 5’ score is also negatively correlated with the expression LFC and the magnitude of correlation improves with increasingly stringent cutoffs (S3A Fig). However, the magnitude of the correlation is not as high as that of PRAS with the 3’ end of 3’ UTR as the reference site (PRAS 3’) (Fig 4A). We also similarly analyzed the targets of CELF1 using PRAS 5’. Again, the PRAS 3’ has stronger negative correlation with the expression LFC than PRAS 5’ for the more reliable CELF1 targets (S3B Fig). The results indicate that known biological knowledge can aid in reference site selection in PRAS for identifying the functional targets of the CELF proteins. The results also suggest that both the CELF4 and CELF1 proteins may regulate mRNAs via the distal 3’ UTRs while having opposite effects on their targets. Indeed, this is plausible because CELF proteins play various roles in both co-transcriptional and post-transcriptional RNA regulation, as well as translation inhibition in different cellular contexts [38–40].

To examine the difference of taking the raw or the normalized read density of the CLIP peaks as the input of PRAS, we then used the Celf4 null iCLIP-seq as the negative control for the wild-type CELF4 iCLIP to score the functional targets of CELF4 with the 3’ end of the 3’UTR as the reference site. Specifically, we replaced the iCLIP-seq read counts *r*_*i*_ in Eq (1) by the enrichment ratio $r_{i}\times {log}_{2}(\frac{r_{i}}{c_{i}})$ as suggested by Van Nostrand et al. [41], where c_i_ is the Celf4 null iCLIP-seq read counts of the ith peak cluster. We noted the PRAS score using the raw read intensity and the enrichment ratio of peaks as PRAS-raw and PRAS-norm, respectively. By applying the correlation analysis as above, we found that PRAS-norm has achieved stronger negative correlation with the expression LFC than PRAS-raw (S4 Fig). This improvement of performance indicates the important role of the negative control in reducing the noise, which is consistent with the results in [42]. Even though PRAS-raw cannot achieve as good performance as PRAS-norm, the difference in the performance between them is small (S4 Fig), which indicates that PRAS can handle the situation where the negative control of CLIP-seq is not available, such as the CELF1 data in our study.

### PRAS identified targets are strongly enriched in functional categories

**Fig 5:**
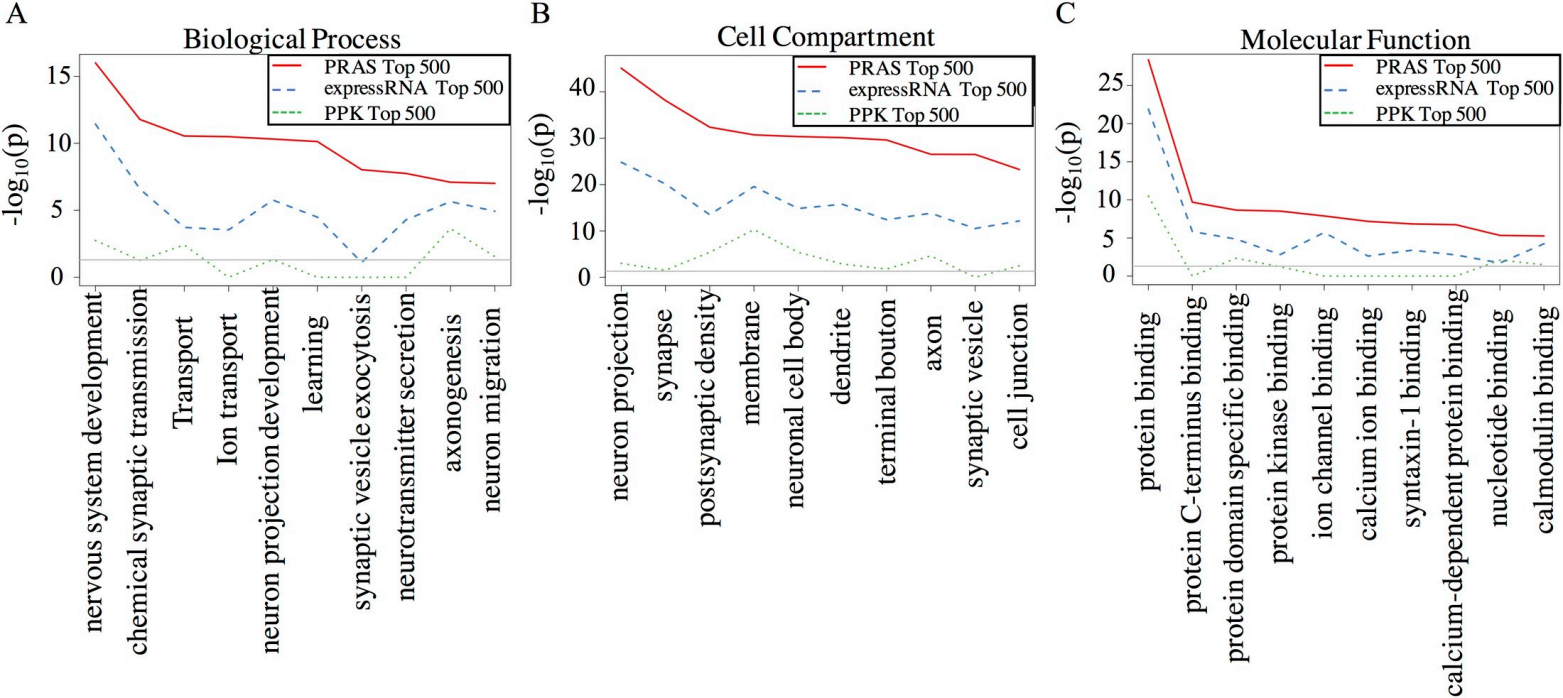
GO analysis of the top ranked targets in different methods and top differentially expressed genes. (A) “Biological Process” GO analysis line-chart. X-axis represents the GO term and Y-axis is the–log_10_(p-value) from the David GO analysis tool (https://david.ncifcrf.gov/) for the top 500 targets ranked by each method. PRAS is highlighted by red solid line, expressRNA is highlighted by blue dashed line, and PPK is highlighted by green dotted line. (B) Similar plot to A but for “Cell Compartment” GO analysis. (C) Similar plot to A but for “Molecular Function” GO analysis.

To further compare the functional relevance of the targets identified by PRAS, expressRNA and PPK, we performed gene ontology (GO) analysis on the top 500 mRNA targets of CELF4 ranked by each score (Fig 5A–5C), which is similar to the analysis shown in Wagnon et al. [29]. There is much greater enrichment (5 to 40 orders based on p-values) of the categories related to suspected CELF4 function in the targets identified by PRAS than those identified by expressRNA and PPK. For example, in the class of “Biological Process”, most of the top 10 significant categories for PRAS top-ranked targets are related to neuron or synaptic functions and ion transport, consistent with prior studies on CELF4 [29]. These results suggest that PRAS captures CELF4 functional targets more precisely than the other methods being compared.

### Functional targets are identified in a large-scale PRAS application to human RBPs

**Fig 6:**
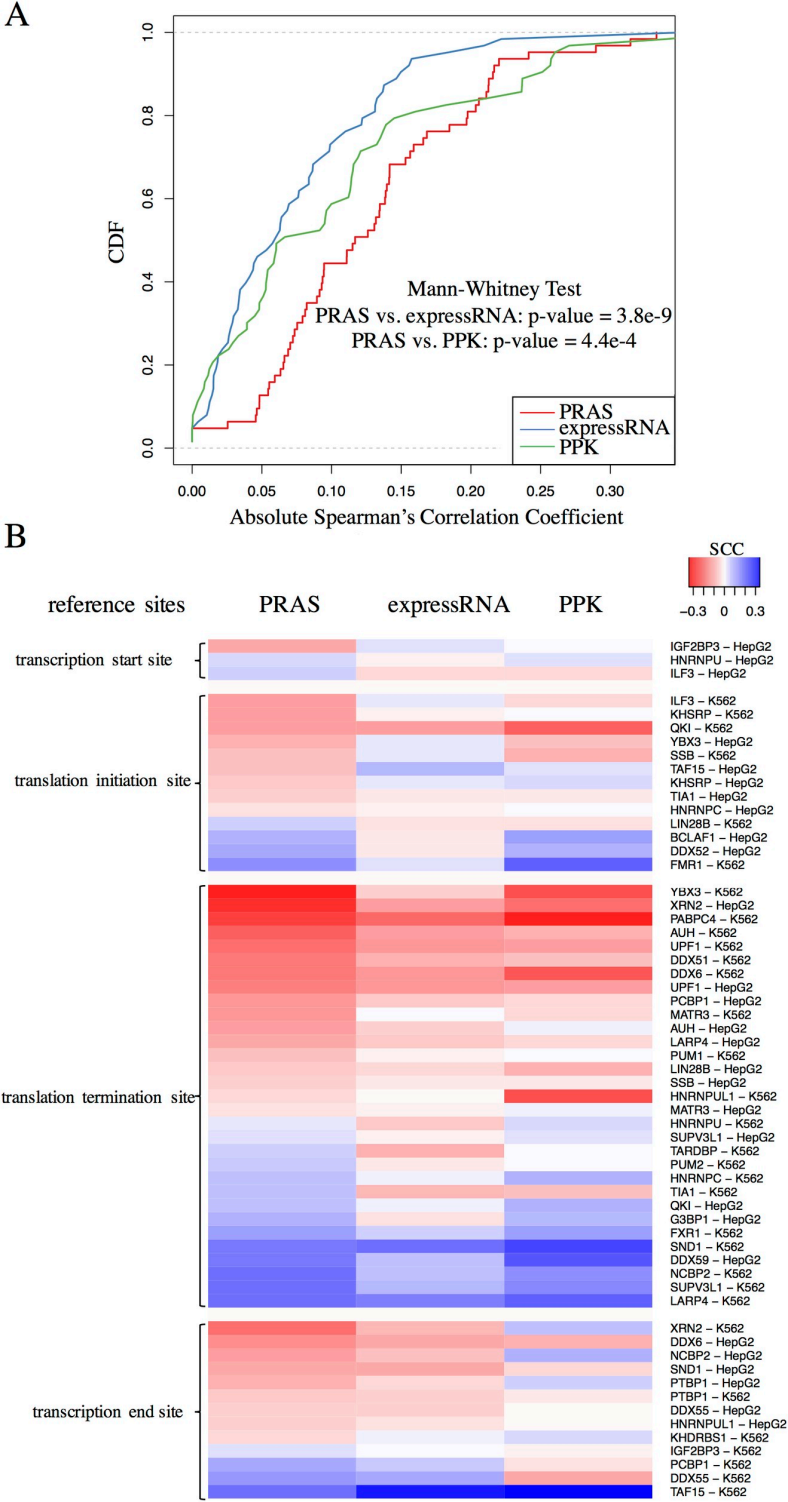
PRAS applied to RNA decay related RBPs. (A) The CDF curve of the absolute correlation coefficient between the gene score and LFC in gene expression. X-axis represents the absolute value of the Spearman’s correlation coefficient between the gene score and LFC in gene expression (KO over wild-type). PRAS, expressRNA, and PPK is highlighted by red, blue and green line, respectively. The p-value of one-sided Mann-Whitney test is listed on the figure. (B) Heatmap of the Spearman’s correlation coefficient. The Spearman’s correlation coefficient between the gene score and LFC in gene expression for PRAS, expressRNA and PPK are listed from the left to the right. The values of the correlation coefficient are indicated by the color, where red and blue color indicates the positive correlation and the negative correlation, respectively. RBPs are grouped by their reference site usage and their ID and cell lines are listed at the right side.

To demonstrate that PRAS has the potential for wide adoption, we further applied PRAS to the eCLIP data [42] in two human cell lines, K562 and HepG2, from the ENCODE consortium [43]. Specifically, we selected the RBPs that are related to the RNA decay function [41] because this function can be clearly quantified at gene level in the differential expression (DE) analysis between the RBP knockdown and the wild-type RNA-seq samples. We collected the DE analysis results by DESeq [44] from ENCODE and obtained 37 distinct RBPs, which include 28 and 32 RBPs in HepG2 and K562 cell line, respectively. We then applied PRAS to the eCLIP data using the enrichment ratio over the control sample described above as the peak intensities. In the parameter settings in PRAS, we selected the reference site for each RBP from 4 candidates: transcription start site, translation initiation site, translation termination site, and transcription end site, based on eCLIP peak intensity distribution along the transcript. S5 Fig presents four example RBPs assigned with 4 different reference sites. To simplify the analysis, we applied *d*_0_ = 500 *nt* to all the selected RBPs according to the distribution (S6 Fig) of the estimated decay parameters as described previously. This general selection of *d*_0_ may not achieve the best performance of PRAS but is likely to be comparable with the best *d*_0_ selection as discussed in the CELF4 data. After obtaining the PRAS scores, we did the correlation analysis of the DE (adjusted p-value < = 0.05) genes for each RBP. We found PRAS scores achieved significantly stronger correlation with the LFC in gene expression in comparison to expressRNA and PPK, with p-value equal to 3.8e-9 and 4.4e-4, respectively (Fig 6A). We then separated the RBPs by their reference site usage and found that the translation termination site and the transcription end site, both of which are related to the 3’ UTR, constitute the majority of the RNA decay regulators’ reference sites (Fig 6B). It suggests the essential association between the 3’ UTR of transcripts and the regulation of their fates by RBPs. In addition, we found that the correlation can reflect important biological functions of RBPs. For example, the 5’ poly(A) site (transcription end site) is used as the reference site for DDX6 in the HepG2 cell line (S5C Fig) and the PRAS score is negatively correlated with the LFC of DDX6’s target gene expression (Fig 6B), which indicates that DDX6 may stabilize its targets via binding near to the poly(A) site. Interestingly, DDX6 is known to be an important regulator in mRNA decapping and degradation [45, 46], which supports our claim that PRAS has the ability to identify the biologically functional targets of the RBP regulators. All these results demonstrate that PRAS has the potential for wide adoption in RBP functional targets identification.

### Discussions on biological insights from the use of PRAS

In this study, we developed PRAS, a position dependent scoring method for identifying and prioritizing RBP functional targets. Weighting the proximity of RBP binding sites to a given reference position exponentially and combining the strengths of the binding signals, we obtained the PRAS scores and the ranking of all the mRNAs that have reliable binding sites of the RBP. We applied this approach to the iCLIP dataset of a neuronal disease-related RBP, CELF4 and to the CLIP dataset of a DM disease-related RBP, CELF1 –both belonging to the CELF family of RBP. We report a much stronger association between CELF4 and its targets at the distal 3’ UTRs compared to internal 3’ UTR positions. We also demonstrate that PRAS performs much better in predicting the mRNA targets stabilized by CELF4, compared to the other existing methods such as expressRNA and PPK. We further observe that PRAS performs much better at predicting the mRNA targets degraded by CELF1. These results not only suggest the importance of incorporating the positional information of the binding sites into target identification, but also suggest the important roles of the distal 3’ UTRs in CELF protein regulated mRNAs.

The binding preferences of RBPs have been noticed in previous studies [3, 29]. However, the link between positional biases of RBP binding sites and their functional consequences has not been well established. PRAS reveals that the distal end of 3’ UTR binding is predictive of CELF4-stabilized targets. The distal end bias of CELF4-stabilized targets suggests possible molecular mechanism(s) by which CELF4 regulates its mRNAs. It has been reported that poly(A) tails enhance the stability of mRNAs [47]. The proximity between poly(A) tails and the distal 3’ UTRs suggests possible connections with poly(A) tail functions, such as mRNA stability, polyadenylation itself or promotion of translational reinitiation–possibilities to be explored in future experimental studies. CELF1 is known to recruit cytoplasmic deadenylases [48] and the extent of mRNA degradation is positively correlated to CELF1’s binding magnitude to the 3’ UTRs [34]. Based on the finding in the previous study [34] that CELF1 binding is enriched in the 3’ end of the 3’ UTR, we further found that this binding bias shows strong predictive ability to CELF1-degraded targets (Fig 3B). We also demonstrated the potential of PRAS in the large-scale applications by showing the better performance of PRAS than other methods in identifying the targets of RNA decay related RBPs from ENCODE [43]. These results again strengthen the relationship between the regulatory functions of the RBPs and their binding positions.

## Availability and future directions

PRAS is implemented in Python and R and is freely available at http://ouyanglab.jax.org/pras/. PRAS can be applied widely to identify the functional targets of any RBPs with CLIP-seq peaks. For RBPs with a known post-transcriptional function, the functional targets may be identified with a corresponding reference position that is related to that function (e.g. splicing sites for alternative splicing). PRAS can also be combined with other types of information, such as sequence motifs, conservation, and perturbation data to predict RBP functional targets using integrative approaches such as [49]. In addition, future versions of PRAS can be extended to study the co-regulations of multiple RBPs by being applied to a set of interested RBPs simultaneously and evaluating the importance of different reference sites on the targets.

## Supporting information

S1 Text Datasets collection. (DOCX)

S1 Fig CELF4 binding characteristics in 3’ UTRs. Shown are distributions of the distances between the iCLIP reads and the proximal/distal end of 3’ UTRs in mRNAs. X-axis represents the distance (number of nucleotide) to the proximal/distal end of 3’ UTRs. Y-axis represents the average iCLIP read counts within the significant peaks at that position across all the genes. The curve for the distal end is highlighted by red color and that for the proximal end is highlighted by blue.
(TIF)

S2 Fig Correlation coefficient curve and AUC curve of PRAS with different *d*_0_s. (A) The line chart of Pearson’s correlation coefficient between the gene score and the gene expression LFC in the qPCR-validated targets of CELF4. The X-axis represents the different *d*_0_s applied to PRAS and the Y-axis shows the value of Pearson’s correlation coefficient. Each dot in the plot is for one *d*_0_ usage in PRAS. (B) Similar to A, but for the AUC values of the ROC analysis. These two line-charts show that the performance of PRAS is stable with the reasonable *d*_0_ selection around 1,000 nt.
(TIF)

S3 Fig Correlation analysis of PRAS with different reference sites. (A) The line chart of Spearman’s correlation coefficient between the gene score and the gene expression LFC in the *Celf4*-regulated list. The X-axis represents the different cutoffs applied to extract the subset of genes and the Y-axis shows the value of Spearman’s correlation coefficient. The corresponding curves for distal PRAS and proximal PRAS are indicated by red and blue lines, respectively. Each dot in the plot is for one subset of genes selected based on the absolute LFC cutoff. (B) Similar to A, but for the *Celf1*-regulated list. These two line-charts show that the top ranked targets by distal PRAS have higher enrichment in the regulated lists comparing to those of proximal PRAS.
(TIF)

S4 Fig Correlation analysis of PRAS with different peak intensity input. The line chart of Spearman’s correlation coefficient between the gene score and the gene expression LFC in the *Celf4*-regulated list. The X-axis represents the different cutoffs applied to extract the subset of genes and the Y-axis shows the value of Spearman’s correlation coefficient. The corresponding curves for PRAS-raw and PRAS-norm are indicated by red and blue lines, respectively. Each dot in the plot is for one subset of genes selected based on the absolute LFC cutoff.
(TIF)

S5 Fig RBP examples of eCLIP signal distribution around different reference sites. (A) Shown are distributions of the distances between the HNRNPU eCLIP peaks and the transcription start site (TSS) in the mRNAs of the HepG2 cell line. X-axis represents the distance (number of nucleotide) to the TSS. Y-axis represents the average eCLIP enrichment ratio within the significant peaks at that position across all the genes. (B) Similar to A, but around the translation initiation site (TIS) for RBP ILF3 in K562 cell line. (C) Similar to A, but around the translation termination site (TTS) for RBP DDX6 in HepG2 cell line. (D) Similar to A, but around the transcription end site (TES) for RBP LARP4 in K562 cell line.
(TIF)

S6 Fig Distribution of the estimated decay parameter for PRAS. Shown are the distributions of the estimated *d*_0_ for PRAS in K562 and HepG2 cell lines. The density curves are highlighted by red and blue for RBPs in K562 and HepG2, respectively. The estimation is done based on the eCLIP peak intensities around the selected reference sites as described in the subsection “PRAS score is a strong predictor of PCR-validated mRNA targets of CELF4”.
(TIF)

## References

[1] Keene JD. RNA regulons: coordination of post-transcriptional events. Nat Rev Genet. 2007;8(7):533–43. 10.1038/nrg2111 17572691

[2] Chenard CA, Richard S. New implications for the QUAKING RNA binding protein in human disease. J Neurosci Res. 2008;86(2):233–42. 10.1002/jnr.21485 17787018

[3] Lebedeva S, Jens M, Theil K, Schwanhausser B, Selbach M, Landthaler M, et al Transcriptome-wide analysis of regulatory interactions of the RNA-binding protein HuR. Mol Cell. 2011;43(3):340–52. 10.1016/j.molcel.2011.06.008 21723171

[4] Yokoshi M, Li Q, Yamamoto M, Okada H, Suzuki Y, Kawahara Y. Direct binding of Ataxin-2 to distinct elements in 3' UTRs promotes mRNA stability and protein expression. Mol Cell. 2014;55(2):186–98. 10.1016/j.molcel.2014.05.022 24954906

[5] Licatalosi DD, Mele A, Fak JJ, Ule J, Kayikci M, Chi SW, et al HITS-CLIP yields genome-wide insights into brain alternative RNA processing. Nature. 2008;456(7221):464–9. 10.1038/nature07488 18978773 PMC2597294

[6] Konig J, Zarnack K, Luscombe NM, Ule J. Protein-RNA interactions: new genomic technologies and perspectives. Nat Rev Genet. 2012;13(2):77–83. 10.1038/nrg3141 22251872

[7] Hafner M, Landthaler M, Burger L, Khorshid M, Hausser J, Berninger P, et al Transcriptome-wide identification of RNA-binding protein and microRNA target sites by PAR-CLIP. Cell. 2010;141(1):129–41. 10.1016/j.cell.2010.03.009 20371350 PMC2861495

[8] Konig J, Zarnack K, Rot G, Curk T, Kayikci M, Zupan B, et al iCLIP reveals the function of hnRNP particles in splicing at individual nucleotide resolution. Nat Struct Mol Biol. 2010;17(7):909–15. 10.1038/nsmb.1838 20601959 PMC3000544

[9] Cook KB, Hughes TR, Morris QD. High-throughput characterization of protein-RNA interactions. Briefings in functional genomics. 2015;14(1):74–89. 10.1093/bfgp/elu047 25504152 PMC4303715

[10] Chen B, Yun J, Kim MS, Mendell JT, Xie Y. PIPE-CLIP: a comprehensive online tool for CLIP-seq data analysis. Genome Biol. 2014;15(1):R18 10.1186/gb-2014-15-1-r18 24451213 PMC4054095

[11] Wang T, Xie Y, Xiao G. dCLIP: a computational approach for comparative CLIP-seq analyses. Genome Biol. 2014;15(1):R11 10.1186/gb-2014-15-1-r11 24398258 PMC4054096

[12] Wang T, Chen B, Kim M, Xie Y, Xiao G. A model-based approach to identify binding sites in CLIP-Seq data. PLoS One. 2014;9(4):e93248 10.1371/journal.pone.0093248 24714572 PMC3979666

[13] Lovci MT, Ghanem D, Marr H, Arnold J, Gee S, Parra M, et al Rbfox proteins regulate alternative mRNA splicing through evolutionarily conserved RNA bridges. Nat Struct Mol Biol. 2013;20(12):1434–42. 10.1038/nsmb.2699 24213538 PMC3918504

[14] Althammer S, Gonzalez-Vallinas J, Ballare C, Beato M, Eyras E. Pyicos: a versatile toolkit for the analysis of high-throughput sequencing data. Bioinformatics. 2011;27(24):3333–40. 10.1093/bioinformatics/btr570 21994224 PMC3232367

[15] Uren PJ, Bahrami-Samani E, Burns SC, Qiao M, Karginov FV, Hodges E, et al Site identification in high-throughput RNA-protein interaction data. Bioinformatics. 2012;28(23):3013–20. 10.1093/bioinformatics/bts569 23024010 PMC3509493

[16] Comoglio F, Sievers C, Paro R. Sensitive and highly resolved identification of RNA-protein interaction sites in PAR-CLIP data. BMC Bioinformatics. 2015;16:32 10.1186/s12859-015-0470-y 25638391 PMC4339748

[17] Corcoran DL, Georgiev S, Mukherjee N, Gottwein E, Skalsky RL, Keene JD, et al PARalyzer: definition of RNA binding sites from PAR-CLIP short-read sequence data. Genome Biol. 2011;12(8):R79 10.1186/gb-2011-12-8-r79 21851591 PMC3302668

[18] Moore MJ, Zhang C, Gantman EC, Mele A, Darnell JC, Darnell RB. Mapping Argonaute and conventional RNA-binding protein interactions with RNA at single-nucleotide resolution using HITS-CLIP and CIMS analysis. Nat Protoc. 2014;9(2):263–93. 10.1038/nprot.2014.012 24407355 PMC4156013

[19] Shah A, Qian Y, Weyn-Vanhentenryck SM, Zhang C. CLIP Tool Kit (CTK): a flexible and robust pipeline to analyze CLIP sequencing data. Bioinformatics. 2017;33(4):566–7. 10.1093/bioinformatics/btw653 27797762 PMC6041811

[20] Erhard F, Dolken L, Jaskiewicz L, Zimmer R. PARma: identification of microRNA target sites in AGO-PAR-CLIP data. Genome Biol. 2013;14(7):R79 10.1186/gb-2013-14-7-r79 23895117 PMC4054675

[21] Modic M, Ule J, Sibley CR. CLIPing the brain: studies of protein-RNA interactions important for neurodegenerative disorders. Mol Cell Neurosci. 2013;56:429–35. 10.1016/j.mcn.2013.04.002 23583633 PMC3793874

[22] Rot G, Wang Z, Huppertz I, Modic M, Lence T, Hallegger M, et al High-Resolution RNA Maps Suggest Common Principles of Splicing and Polyadenylation Regulation by TDP-43. Cell Rep. 2017;19(5):1056–67. 10.1016/j.celrep.2017.04.028 28467899 PMC5437728

[23] Mukherjee N, Corcoran DL, Nusbaum JD, Reid DW, Georgiev S, Hafner M, et al Integrative regulatory mapping indicates that the RNA-binding protein HuR couples pre-mRNA processing and mRNA stability. Mol Cell. 2011;43(3):327–39. 10.1016/j.molcel.2011.06.007 21723170 PMC3220597

[24] Meins M, Schlickum S, Wilhelm C, Missbach J, Yadav S, Glaser B, et al Identification and characterization of murine Brunol4, a new member of the elav/bruno family. Cytogenet Genome Res. 2002;97(3–4):254–60. 10.1159/000066619 12438720

[25] Yang Y, Mahaffey CL, Berube N, Maddatu TP, Cox GA, Frankel WN. Complex seizure disorder caused by Brunol4 deficiency in mice. PLoS Genet. 2007;3(7):e124 10.1371/journal.pgen.0030124 17677002 PMC1934399

[26] Halgren C, Bache I, Bak M, Myatt MW, Anderson CM, Brondum-Nielsen K, et al Haploinsufficiency of CELF4 at 18q12.2 is associated with developmental and behavioral disorders, seizures, eye manifestations, and obesity. European journal of human genetics: EJHG. 2012;20(12):1315–9. 10.1038/ejhg.2012.92 22617346 PMC3499750

[27] Barone R, Fichera M, De Grandi M, Battaglia M, Lo Faro V, Mattina T, et al Familial 18q12.2 deletion supports the role of RNA-binding protein CELF4 in autism spectrum disorders. American journal of medical genetics Part A. 2017;173(6):1649–55. 10.1002/ajmg.a.38205 28407444

[28] Wagnon JL, Mahaffey CL, Sun W, Yang Y, Chao HT, Frankel WN. Etiology of a genetically complex seizure disorder in Celf4 mutant mice. Genes, brain, and behavior. 2011;10(7):765–77. 10.1111/j.1601-183X.2011.00717.x 21745337 PMC3190060

[29] Wagnon JL, Briese M, Sun W, Mahaffey CL, Curk T, Rot G, et al CELF4 regulates translation and local abundance of a vast set of mRNAs, including genes associated with regulation of synaptic function. PLoS Genet. 2012;8(11):e1003067 10.1371/journal.pgen.1003067 23209433 PMC3510034

[30] Timchenko LT, Timchenko NA, Caskey CT, Roberts R. Novel proteins with binding specificity for DNA CTG repeats and RNA CUG repeats: implications for myotonic dystrophy. Hum Mol Genet. 1996;5(1):115–21. 10.1093/hmg/5.1.115 8789448

[31] Kalsotra A, Xiao X, Ward AJ, Castle JC, Johnson JM, Burge CB, et al A postnatal switch of CELF and MBNL proteins reprograms alternative splicing in the developing heart. Proc Natl Acad Sci U S A. 2008;105(51):20333–8. 10.1073/pnas.0809045105 19075228 PMC2629332

[32] Ladd AN, Stenberg MG, Swanson MS, Cooper TA. Dynamic balance between activation and repression regulates pre-mRNA alternative splicing during heart development. Dev Dyn. 2005;233(3):783–93. 10.1002/dvdy.20382 15830352

[33] Moraes JC, Amaral ME, Picardi PK, Calegari VC, Romanatto T, Bermudez-Echeverry M, et al Inducible-NOS but not neuronal-NOS participate in the acute effect of TNF-alpha on hypothalamic insulin-dependent inhibition of food intake. FEBS Lett. 2006;580(19):4625–31. 10.1016/j.febslet.2006.07.042 16876161

[34] Wang ET, Ward AJ, Cherone JM, Giudice J, Wang TT, Treacy DJ, et al Antagonistic regulation of mRNA expression and splicing by CELF and MBNL proteins. Genome Res. 2015;25(6):858–71. 10.1101/gr.184390.114 25883322 PMC4448682

[35] Ouyang Z, Zhou Q, Wong WH. ChIP-Seq of transcription factors predicts absolute and differential gene expression in embryonic stem cells. Proc Natl Acad Sci U S A. 2009;106(51):21521–6. 10.1073/pnas.0904863106 19995984 PMC2789751

[36] Kent WJ, Sugnet CW, Furey TS, Roskin KM, Pringle TH, Zahler AM, et al The human genome browser at UCSC. Genome Res. 2002;12(6):996–1006. 10.1101/gr.229102 12045153 PMC186604

[37] Quinlan AR, Hall IM. BEDTools: a flexible suite of utilities for comparing genomic features. Bioinformatics. 2010;26(6):841–2. 10.1093/bioinformatics/btq033 20110278 PMC2832824

[38] Dasgupta T, Ladd AN. The importance of CELF control: molecular and biological roles of the CUG-BP, Elav-like family of RNA-binding proteins. Wiley Interdiscip Rev RNA. 2012;3(1):104–21. 10.1002/wrna.107 22180311 PMC3243963

[39] Mukhopadhyay D, Houchen CW, Kennedy S, Dieckgraefe BK, Anant S. Coupled mRNA stabilization and translational silencing of cyclooxygenase-2 by a novel RNA binding protein, CUGBP2. Mol Cell. 2003;11(1):113–26. 12535526 10.1016/s1097-2765(03)00012-1

[40] Subramaniam D, Natarajan G, Ramalingam S, Ramachandran I, May R, Queimado L, et al Translation inhibition during cell cycle arrest and apoptosis: Mcl-1 is a novel target for RNA binding protein CUGBP2. Am J Physiol Gastrointest Liver Physiol. 2008;294(4):G1025–32. 10.1152/ajpgi.00602.2007 18292181

[41] Van Nostrand EL, Freese P, Pratt GA, Wang X, Wei X, Xiao R, et al A Large-Scale Binding and Functional Map of Human RNA Binding Proteins. bioRxiv. 2018:179648. 10.1038/s41586-020-2077-3 PMC7410833 32728246

[42] Van Nostrand EL, Pratt GA, Shishkin AA, Gelboin-Burkhart C, Fang MY, Sundararaman B, et al Robust transcriptome-wide discovery of RNA-binding protein binding sites with enhanced CLIP (eCLIP). Nat Methods. 2016;13(6):508–14. 10.1038/nmeth.3810 27018577 PMC4887338

[43] Consortium EP. An integrated encyclopedia of DNA elements in the human genome. Nature. 2012;489(7414):57–74. 10.1038/nature11247 22955616 PMC3439153

[44] Love MI, Huber W, Anders S. Moderated estimation of fold change and dispersion for RNA-seq data with DESeq2. Genome Biol. 2014;15(12):550 10.1186/s13059-014-0550-8 25516281 PMC4302049

[45] Fenger-Gron M, Fillman C, Norrild B, Lykke-Andersen J. Multiple processing body factors and the ARE binding protein TTP activate mRNA decapping. Mol Cell. 2005;20(6):905–15. 10.1016/j.molcel.2005.10.031 16364915

[46] Hu G, McQuiston T, Bernard A, Park YD, Qiu J, Vural A, et al A conserved mechanism of TOR-dependent RCK-mediated mRNA degradation regulates autophagy. Nat Cell Biol. 2015;17(7):930–42. 10.1038/ncb3189 26098573 PMC4528364

[47] Subtelny AO, Eichhorn SW, Chen GR, Sive H, Bartel DP. Poly(A)-tail profiling reveals an embryonic switch in translational control. Nature. 2014;508(7494):66–71. 10.1038/nature13007 24476825 PMC4086860

[48] Vlasova-St Louis I, Dickson AM, Bohjanen PR, Wilusz CJ. CELFish ways to modulate mRNA decay. Biochim Biophys Acta. 2013;1829(6–7):695–707. 10.1016/j.bbagrm.2013.01.001 23328451 PMC3640684

[49] Zhang C, Frias MA, Mele A, Ruggiu M, Eom T, Marney CB, et al Integrative modeling defines the Nova splicing-regulatory network and its combinatorial controls. Science. 2010;329(5990):439–43. 10.1126/science.1191150 20558669 PMC3412410

