# Supplementary material for: PRAS: Predicting functional targets of RNA binding proteins based on CLIP-seq peaks
Source: PLoS Comput Biol. 2019 Aug 19;15(8):e1007227. doi: 10.1371/journal.pcbi.1007227 (PMC6716675; doi:10.1371/journal.pcbi.1007227)
Supplement: S1 Text — (DOCX) [file pcbi.1007227.s001.docx]

**Data collection**

**CLIP-seq datasets**

The iCLIP dataset and cross-linking sites of CELF4 in mouse brains were generated and processed previously [1]. Our analysis focuses on cross-linking sites detected by iCount (http://icount.fri.uni-lj.si) [2] with FDR less than or equal to 0.05. The annotation of 3’ UTRs was downloaded from the RefSeq mm9 track in the UCSC table browser (https://genome.ucsc.edu/) [3]. If the location of a cross-linking site can be assigned to more than one 3’ UTR, then the nearest distal 3’ UTR annotation was used for calculation. The CLIP-seq dataset of CELF1 in mouse muscle were generated and process previously [4]. Our analysis used the called peaks from their paper and used the same annotation of 3’ UTRs as that for CELF4 datasets.

**qPCR dataset**

The qPCR dataset was generated previously [1] in cortex and hippocampus of the mouse brain. We extracted the genes with p-value less or equal to 0.1 in each tissue as the validated targets, where the p-values were calculated using the Student’s t-test and reported in Wagnon et al [1]. We constructed the qPCR-validated dataset by combining the validated targets from the two tissues and excluding two genes that are not annotated in Refseq, which resulted in 23 mRNA targets in total. For the 4 targets in the qPCR-validated dataset that were examined in both tissues, as they had the same trend of expression change in the mutants, their LFC were calculated as the average LFC of the two tissues extracted from Wagnon et al. [1].

**Microarray and RNA-seq datasets**

We used the wild-type and *Celf4* null mouse brain microarray datasets performed and compiled by Wagnon et al [1]. There are three samples each in the wild-type and *Celf4* null mouse brains. We extracted the permutation p-values provided by MAANOVA (<http://churchill.jax.org/software/jmaanova.shtml)> [5]. We also obtained the log fold change of the qPCR values in *Celf4* null over mouse brains. We used the wild-type and *Celf1* over-expression mouse muscle RNA-seq datasets from Wang et al [4]. We used DESeq [6] to analyze the differential expression of genes and calculated the adjusted p-values using the Benjamini and Hochberg method [7].

**ENCODE eCLIP and RNA-seq datasets**

We used the narrow peak file of RBPs generated from the eCLIP datasets in ENCODE [8]. The RBPs with RNA decay functions were compiled by Van Nostrand et al [9]. Differential expression (DE) analysis results between the wild-type and knock-out RNA-seq datasets were collected from ENCODE. We used 0.05 as the adjusted p-value equal cutoff to define the significantly DE genes.

Reference:

1. Wagnon JL, Briese M, Sun W, Mahaffey CL, Curk T, Rot G, et al. CELF4 regulates translation and local abundance of a vast set of mRNAs, including genes associated with regulation of synaptic function. PLoS Genet. 2012;8(11):e1003067.

2. Curk T, Rot G, C. G, J. Z, Konig J, Y. S, et al. iCount: protein-RNA interaction iCLIP data analysis. 2016.

3. Karolchik D, Hinrichs AS, Furey TS, Roskin KM, Sugnet CW, Haussler D, et al. The UCSC Table Browser data retrieval tool. Nucleic Acids Res. 2004;32(Database issue):D493-6.

4. Wang ET, Ward AJ, Cherone JM, Giudice J, Wang TT, Treacy DJ, et al. Antagonistic regulation of mRNA expression and splicing by CELF and MBNL proteins. Genome Res. 2015;25(6):858-71.

5. Wu H, Yang m, KSwifG. C, K. K, Cui X. maanova: Tools for analyzing Micro Array experiments. 2018.

6. Love MI, Huber W, Anders S. Moderated estimation of fold change and dispersion for RNA-seq data with DESeq2. Genome Biol. 2014;15(12):550.

7. Benjamini Y, Y. H. Controlling the False Discovery Rate: A Practical and Powerful Approach to Multiple Testing. Journal of the Royal Statistical Society. 1995;57(1):12.

8. Consortium EP. An integrated encyclopedia of DNA elements in the human genome. Nature. 2012;489(7414):57-74.

9. Van Nostrand EL, Freese P, Pratt GA, Wang X, Wei X, Xiao R, et al. A Large-Scale Binding and Functional Map of Human RNA Binding Proteins. bioRxiv. 2018:179648.
