# Supplementary figures and images for: PRAS: Predicting functional targets of RNA binding proteins based on CLIP-seq peaks
Source: PLoS Comput Biol. 2019 Aug 19;15(8):e1007227. doi: 10.1371/journal.pcbi.1007227 (PMC6716675; doi:10.1371/journal.pcbi.1007227)

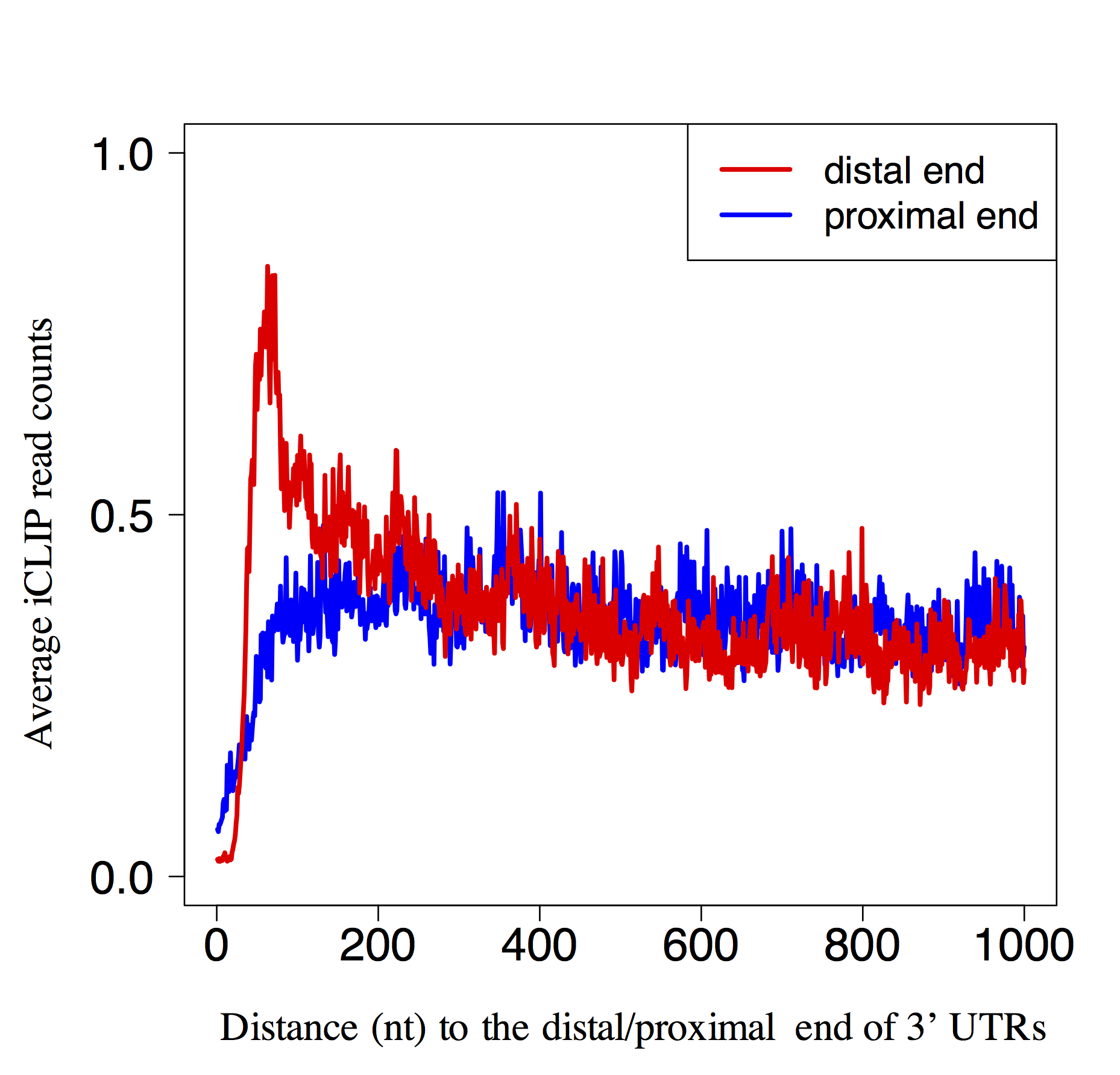

Supplement: S1 Fig — Shown are distributions of the distances between the iCLIP reads and the proximal/distal end of 3’ UTRs in mRNAs. X-axis represents the distance (number of nucleotide) to the proximal/distal end of 3’ UTRs. Y-axis represents the average iCLIP read counts within the significant peaks at that position across all the genes. The curve for the distal end is highlighted by red color and that for the proximal end is highlighted by blue. (TIF) [file pcbi.1007227.s002.tif]

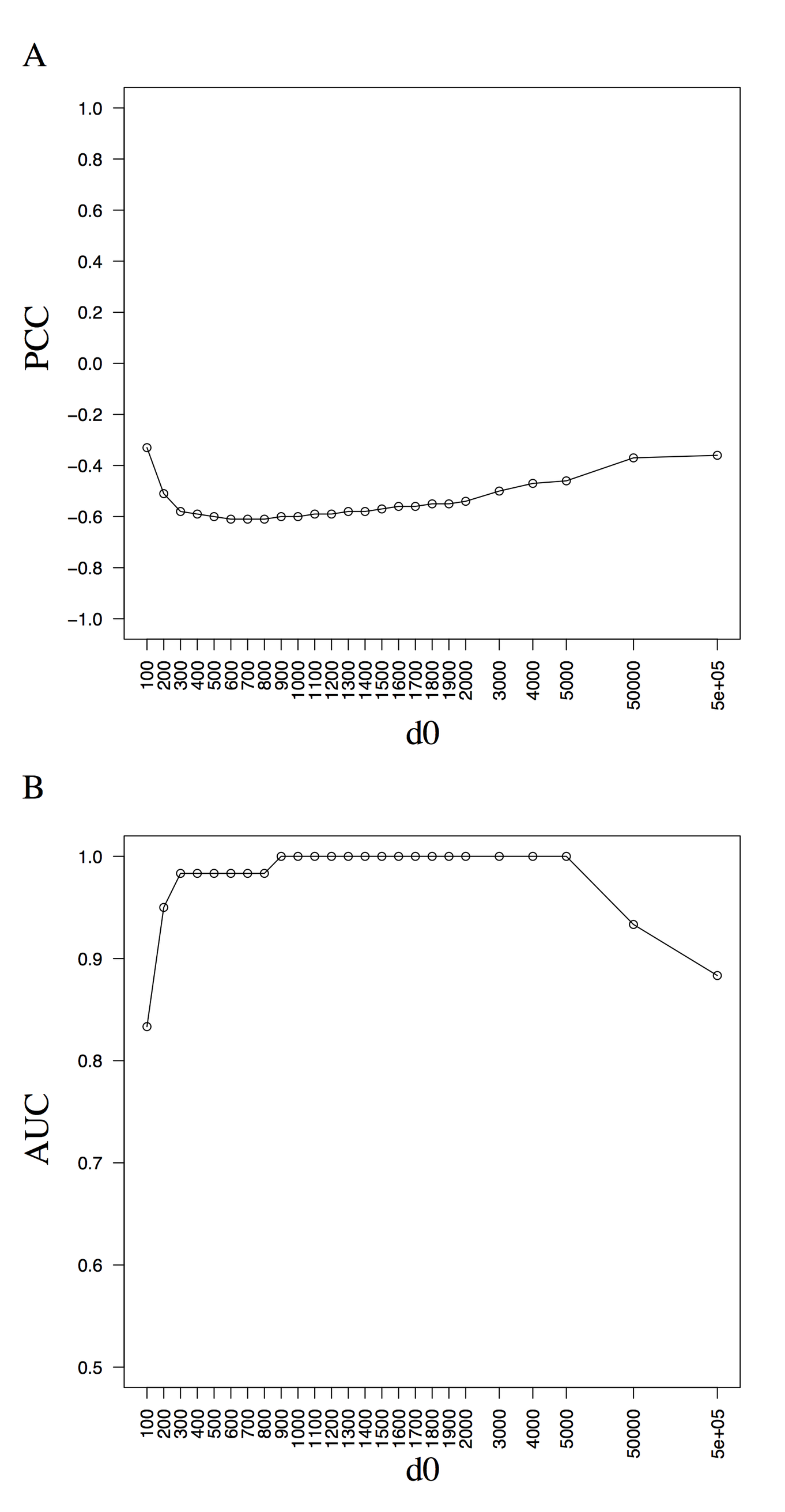

Supplement: S2 Fig — (A) The line chart of Pearson’s correlation coefficient between the gene score and the gene expression LFC in the qPCR-validated targets of CELF4. The X-axis represents the different d0s applied to PRAS and the Y-axis shows the value of Pearson’s correlation coefficient. Each dot in the plot is for one d0 usage in PRAS. (B) Similar to A, but for the AUC values of the ROC analysis. These two line-charts show that the performance of PRAS is stable with the reasonable d0 selection around 1,000 nt. (TIF) [file pcbi.1007227.s003.tif]

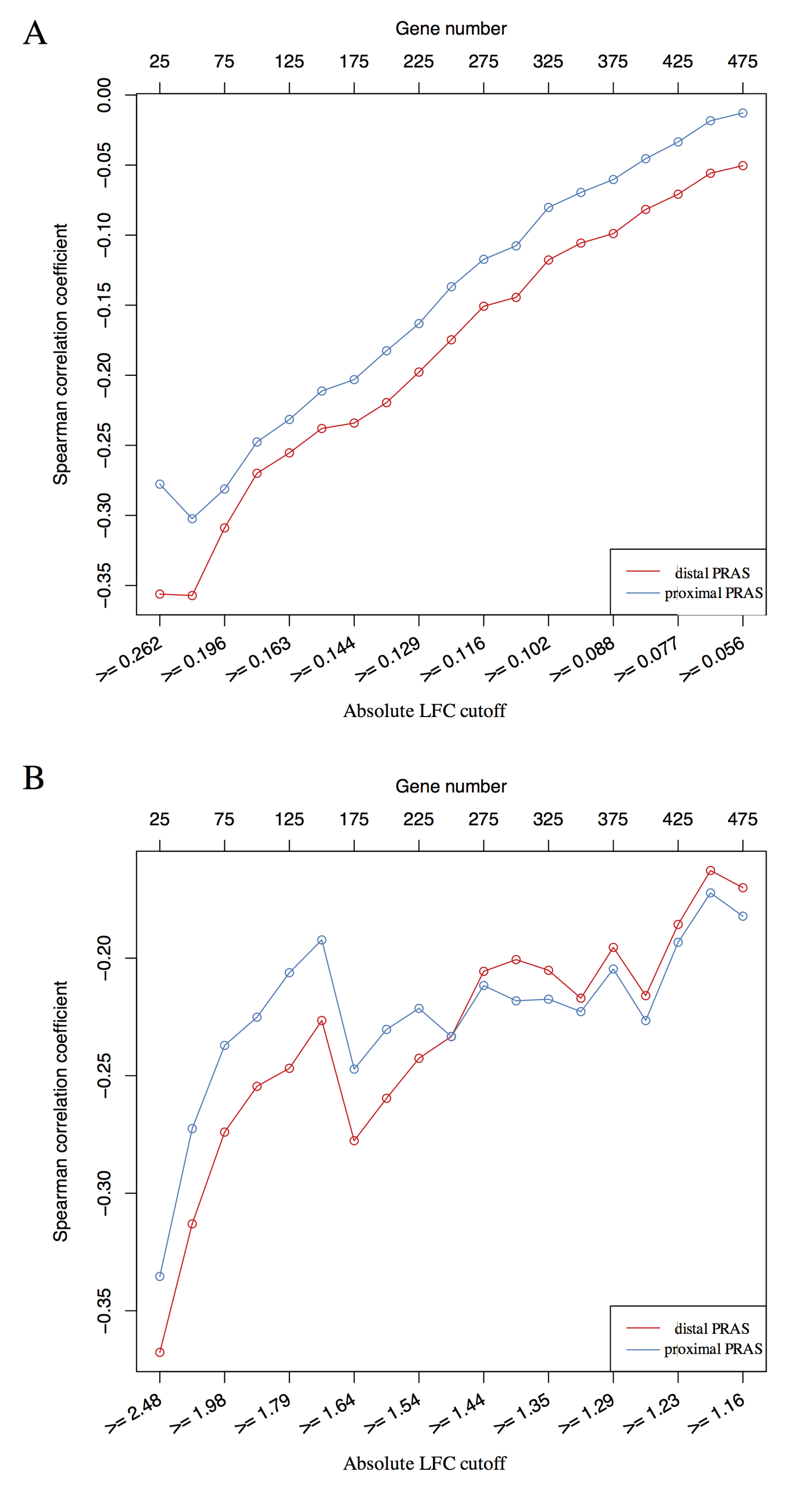

Supplement: S3 Fig — (A) The line chart of Spearman’s correlation coefficient between the gene score and the gene expression LFC in the Celf4-regulated list. The X-axis represents the different cutoffs applied to extract the subset of genes and the Y-axis shows the value of Spearman’s correlation coefficient. The corresponding curves for distal PRAS and proximal PRAS are indicated by red and blue lines, respectively. Each dot in the plot is for one subset of genes selected based on the absolute LFC cutoff. (B) Similar to A, but for the Celf1-regulated list. These two line-charts show that the top ranked targets by distal PRAS have higher enrichment in the regulated lists comparing to those of proximal PRAS. (TIF) [file pcbi.1007227.s004.tif]

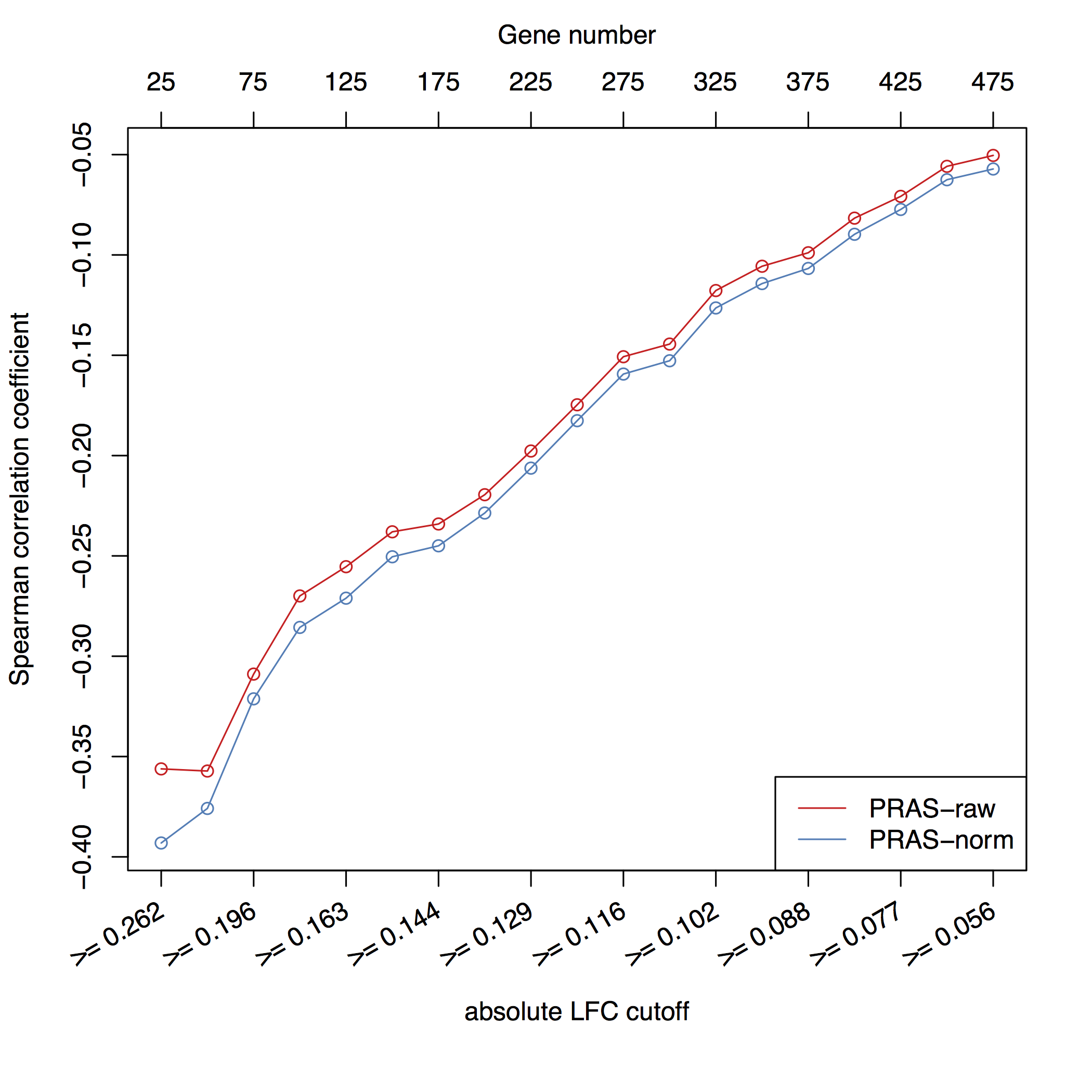

Supplement: S4 Fig — The line chart of Spearman’s correlation coefficient between the gene score and the gene expression LFC in the Celf4-regulated list. The X-axis represents the different cutoffs applied to extract the subset of genes and the Y-axis shows the value of Spearman’s correlation coefficient. The corresponding curves for PRAS-raw and PRAS-norm are indicated by red and blue lines, respectively. Each dot in the plot is for one subset of genes selected based on the absolute LFC cutoff. (TIF) [file pcbi.1007227.s005.tif]

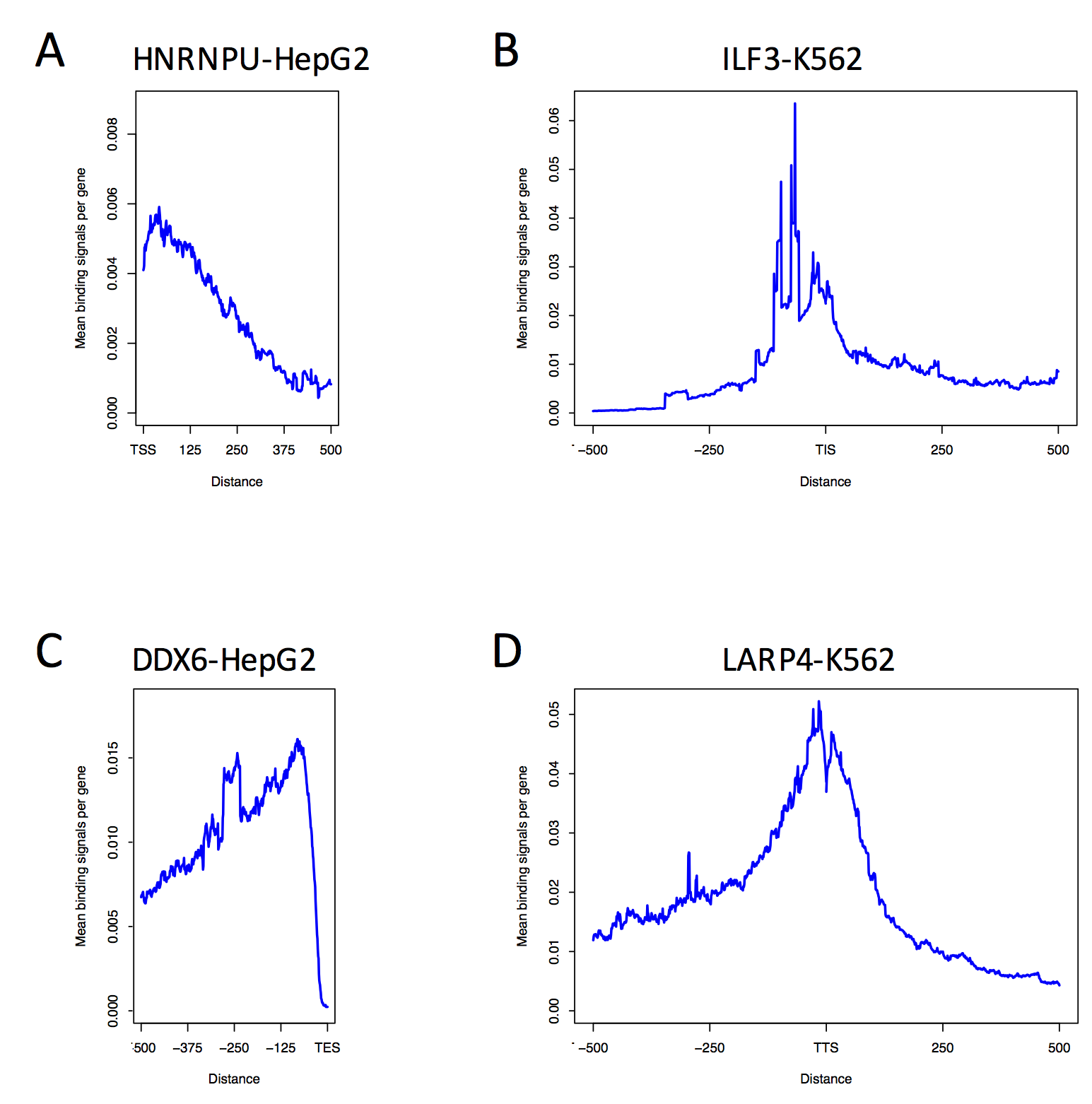

Supplement: S5 Fig — (A) Shown are distributions of the distances between the HNRNPU eCLIP peaks and the transcription start site (TSS) in the mRNAs of the HepG2 cell line. X-axis represents the distance (number of nucleotide) to the TSS. Y-axis represents the average eCLIP enrichment ratio within the significant peaks at that position across all the genes. (B) Similar to A, but around the translation initiation site (TIS) for RBP ILF3 in K562 cell line. (C) Similar to A, but around the translation termination site (TTS) for RBP DDX6 in HepG2 cell line. (D) Similar to A, but around the transcription end site (TES) for RBP LARP4 in K562 cell line. (TIF) [file pcbi.1007227.s006.tif]

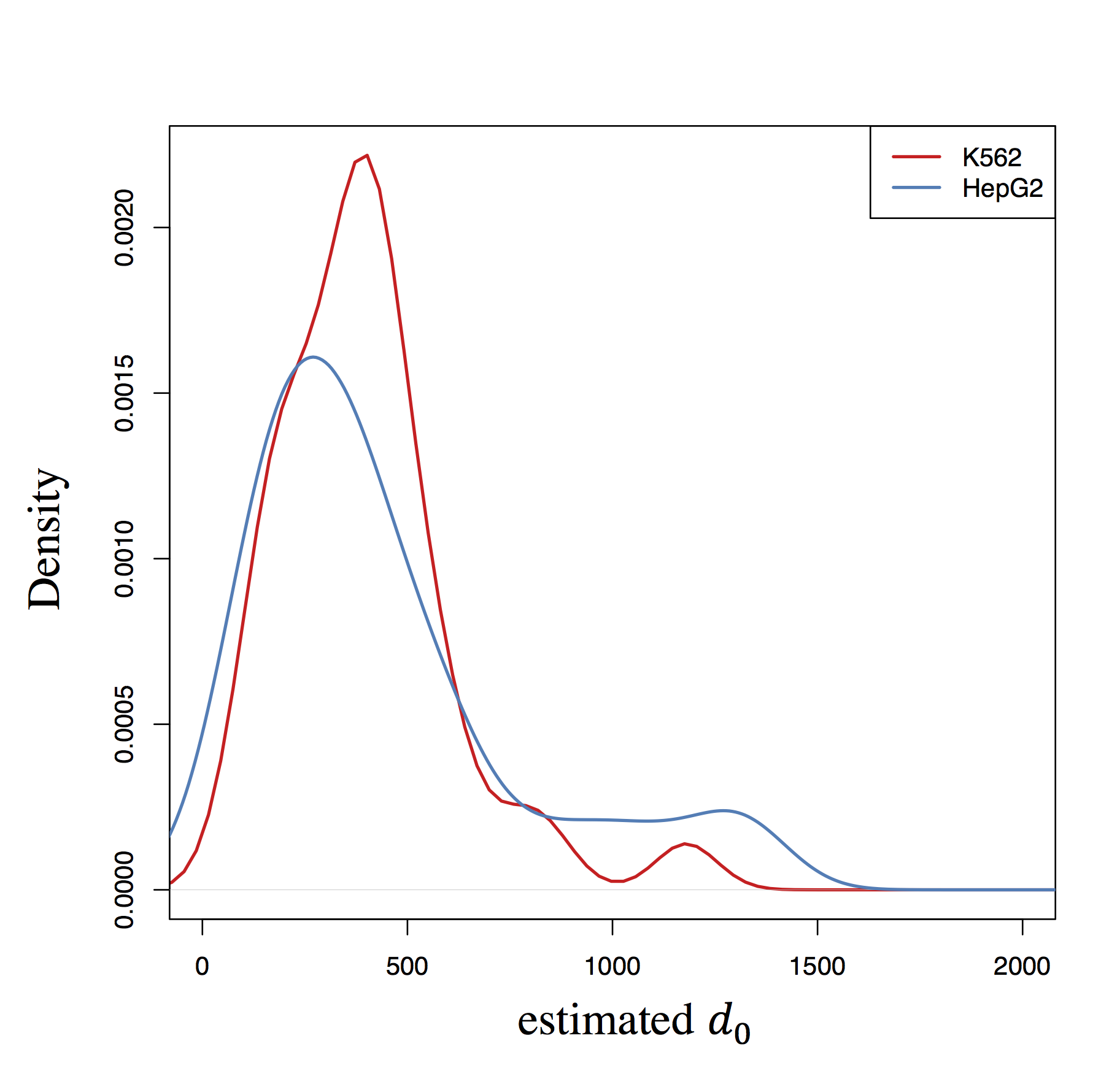

Supplement: S6 Fig — Shown are the distributions of the estimated d0 for PRAS in K562 and HepG2 cell lines. The density curves are highlighted by red and blue for RBPs in K562 and HepG2, respectively. The estimation is done based on the eCLIP peak intensities around the selected reference sites as described in the subsection “PRAS score is a strong predictor of PCR-validated mRNA targets of CELF4”. (TIF) [file pcbi.1007227.s007.tif]
